# Dia-B-Ties: B Cells in the Islet–Immune-Cell Interface in T1D

**DOI:** 10.3390/biom15030332

**Published:** 2025-02-25

**Authors:** Brandon K. Hilliard, Jessica E. Prendergast, Mia J. Smith

**Affiliations:** 1 Barbara Davis Center for Diabetes, University of Colorado School of Medicine, Aurora, CO 80045, USA; 2 Department of Immunology and Microbiology, University of Colorado School of Medicine, Aurora, CO 80045, USA

**Keywords:** B cells, B lymphocytes, type 1 diabetes, autoantibodies, antigen-presenting cells, insulitis

## Abstract

Type 1 diabetes (T1D) is an autoimmune disease that affects an estimated 30 million people worldwide and results in a lifelong dependency of exogenous insulin treatments. While T1D is characterized by T-cell driven-destruction of the insulin-secreting β cells, B lymphocytes play a key role in the islet–immune interface. B cells are an essential intermediary between islet cells and other immune-cell populations. Through antigen presentation, cytokine secretion, and antibody production, B cells play a role in activating autoreactive islet-specific T cells, thus potentiating pancreatic inflammation in the early stages of T1D. Despite this, their role in disease development remains an understudied feature of T1D with significant therapeutic potential. Herein, we will discuss the current knowledge of the islet–immune-cell interface within T1D through the lens of B lymphocytes. We will also consider knowledge gaps that may be limiting further therapeutic opportunities.

## 1. Introduction

Type 1 diabetes (T1D) is an autoimmune disease defined by the destruction of insulin-producing β cells of the pancreas, resulting in dysglycemia and lifelong dependence on exogenous insulin. Despite significant improvements in the treatment of dysglycemia, thanks to continuous glucose monitors and automated insulin pumps, there remains no cure for T1D [1]. Indeed, even with optimal glucose regulation, patients with T1D remain at high risk for disease-associated mortality and co-morbidities, such as retinopathy, nephropathy, and cardiovascular disease [2]. Furthermore, increases in disease incidence and the cost of treatment over the last decade have only increased the urgency to identify preventative and disease modifying interventions. T1D is a complex disease with various immune cells that contribute to the disease. T cells are of particular interest in this regard. Indeed, β-cell destruction is primarily driven by cytotoxic CD8+ T cells and aided by CD4+ T helper cells [1]. However, B cells are also an essential contributor to disease development through the activation of autoreactive T cells and potentiating local inflammation in the pancreas [3].

In this review, we will explore T1D pathogenesis from the perspective of B cells. Specifically, we will discuss how the three main effector functions of B cells, antibody production, antigen presentation, and cytokine secretion, each contribute uniquely to local pathology. Then, we will explore the pancreatic architecture in humans versus mice and how this architecture contributes to B-cell infiltration and associated inflammation. Next, we will discuss the timing, role, and localization of B-cell infiltration during disease progression in relation to disease endotypes. Finally, we will discuss current B-cell-targeting therapies, including their mechanisms of action, rationale, and effectiveness in late-phase clinical trials.

## 2. The Role of B Cells in T1D and How They Interact with Other Immune Cells

### 2.1. The Case for B Cells in Type 1 Diabetes

It is well established that T1D is caused by the destruction of insulin-producing β cells of the pancreas. This process is primarily driven by CD8+ T cells and assisted by CD4+ helper T cells [4]. Despite this, increasing evidence points to an essential role for B cells in T1D pathogenesis. Evidence for the key roles of B cells is supported by various studies in both mice and humans. In the non-obese diabetic (NOD) mouse, which develops spontaneous autoimmune diabetes, B cells are required for disease development [5]. Furthermore, early depletion of B cells using anti-CD20 results in the attenuation of disease [6]. Additionally, results from a Phase 2 clinical trial using rituximab in new-onset T1D patients showed that patients treated with the B-cell-depleting anti-CD20 monoclonal antibody exhibited preserved C-peptide and a decreased exogenous insulin dosage compared to placebo-treated patients one year after therapy [6,7]. Lastly, while T1D is a disease of homogenous symptoms, there is increasing evidence that supports the idea of heterogeneous etiologies. Specifically, the concept of T1D endotypes suggests that islet-reactive B cells may play a more pathogenic role in those that develop T1D at an early age. This concept stems from the fact that these young-onset individuals have increased numbers of B cells in their pancreas compared to older-onset patients [8,9]. Taken together, these observations support an important role for B cells in the development of T1D.

**Figure 1:**
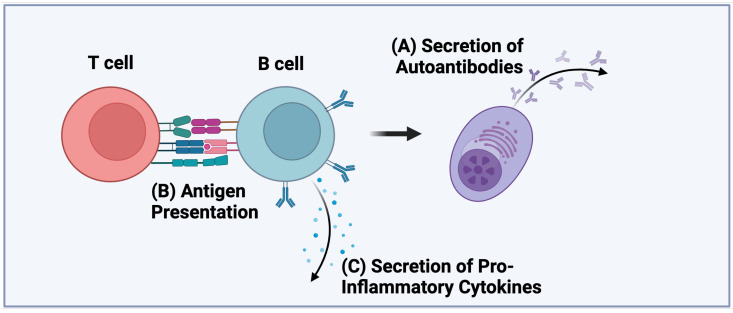
Effector functions of B cells in T1D pathogenesis. (**A**) B Antigen to CD4+ and CD8+ T cells, (**B**) autoantibody production, and (**C**) cytokine production by B cells.

In healthy individuals, self-reactive B cells are normally tolerized centrally in the bone marrow through receptor editing or clonal deletion, or peripherally through anergy. It has been demonstrated that individuals with T1D exhibit defects in both central and peripheral tolerance that allows the escape of self-reactive B cells into the periphery unimpeded [10,11]. Once out in the periphery, B cells participate in the development of T1D via three primary effector functions, antigen presentation, autoantibody production, and cytokine production, which are discussed in more detail below (Figure 1). Given that the development of T1D is driven to some extent by genetic risk alleles, it seems plausible that these factors contribute to the loss of central and peripheral tolerance and the ability of autoreactive B cells to conduct their various effector functions. In line with this, it has been demonstrated that the loss of anergic B cells from the peripheral blood is associated with the high-risk HLA DR4-DQ8 haplotype [12]. In addition, non-HLA genetic risk alleles in PTPN2, PTPN22, INS, and IKZF3 have also been shown to be associated with the loss of B-cell tolerance [12]. While it has not been extensively studied, other T1D-associated allelic variants of genes that are expressed in B cells, such as BACH2, SH2B3, CTSH, and IL10, may also impact B-cell tolerance and the ability of self-reactive B cells to become activated and participate in the development of T1D. Future studies are needed to address this possibility. In the following section, we will discuss each of the B-cell effector functions and consider their contribution to T1D pathogenesis.

### 2.2. Autoantibody Production by B Cells in T1D

**Figure 2:**
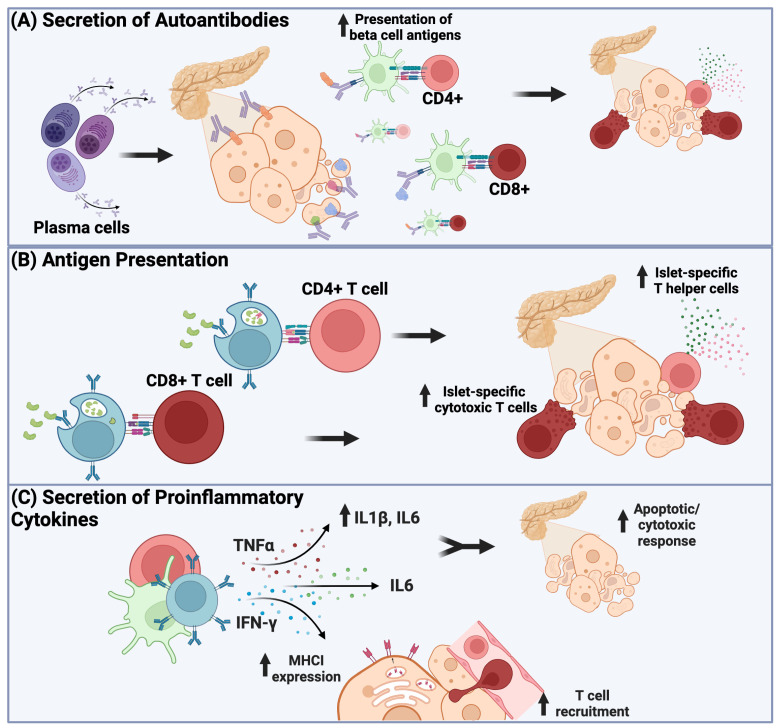
Pathological function of B cells in T1D development. (**A**) Autoantibodies, while not the primary drivers of β cell destruction, also have the capacity to potentiate β cell destruction. Through Fc receptors on the cell surface of professional antigen-presenting cells (APCs), such as dendritic cells and macrophages, APCs can enhance the activation of autoreactive T cells through Fc-mediated uptake of islet antigen–antibody complexes that are, in turn, presented to self-reactive T cells. (**B**) B cells can present antigen bound by their respective BCRs to CD4 T cells. Additionally, B cells can present antigen to CD8 T cells through cross-presentation. Following activation by B cells, CD4 and CD8 T cells are the primary effector cells in pancreatic β-cell destruction. (**C**) B cells can produce various pro-inflammatory cytokines, including TNFα, IL1β, IL6, and IFN-γ. These cytokines contribute to T1D pathogenesis by increasing MHC I expression on the surface of β cells, enhancing recruitment of T cells to the site of inflammation (i.e., pancreas), and contributing to an increased apoptotic response of β cells.

One of the best well-known functions of B cells is their ability to differentiate into antibody-secreting cells, such as plasmablasts and plasma cells (Figure 2A). In T1D, the autoantibodies produced by self-reactive B cells are used to identify individuals at risk for the development of T1D and diagnose someone with T1D. The most commonly assayed autoantibodies include those recognizing insulin, glutamic acid decarboxylase (65 kDa) (GAD65), islet antigen-2 (IA-2), and zinc transporter 8 (ZnT8). Currently, T1D can be divided into different stages that begin prior to a diagnosis of T1D. Stage 1 T1D is someone who has at least two autoantibodies but is normoglycemic, Stage 2 T1D occurs when a person has two or more autoantibodies and shows signs of dysglycemia, and Stage 3 is a clinical diagnosis of T1D requiring exogenous insulin administration. Despite many attempts to identify a better biomarker for increased risk for the development of T1D, the detection of autoantibodies is currently the best biomarker available. However, it is less clear whether autoantibodies play a more important role in the pathogenesis of T1D other than as an indicator that the autoimmune process has begun. Classic dogma suggests that islet antigen-reactive antibodies, while essential biomarkers of disease, do not play a pathological role in disease development. This notion is supported by the following observations: First, purified IgG transferred from diabetic NOD mice is not sufficient to induce disease in B-cell-deficient mice [13], nor does the direct transfer of human serum antibodies induce diabetes in NODScid mice [14]. In addition, the maternal transfer of autoantibodies to the fetus does not increase the risk of T1D in offspring [15]. Furthermore, Wong et al. showed that in mlg mice, a mouse model in which B cells express IgM on the B-cell surface but are unable to secrete antibody, the development of autoimmune diabetes can still occur [16]. While these studies demonstrate that antibody production is not essential for disease development, they do not exclude a role for islet-reactive antibodies in the potentiation of the disease.

Evidence supporting a role for antibodies in disease development stems from the observation that NOD mice with Fc receptor deficiencies are less prone to disease development [17]. Both FcR common γ-chain knock-out (KO) and FcγRIII KO NOD mice exhibit a decrease in disease incidence and an increase in average time of onset compared to wild-type (WT) NOD mice [17]. Interestingly, disease incidence was rescued when WT NOD dendric cells (DCs) or natural killer (NK) cells were transferred into FcR common γ-chain KO mice, demonstrating that the ability of DCs and NK cells to engage antibodies is a likely mediator of disease incidence [17]. The acquisition of antigen–antibody complexes through Fc receptors on antigen-presenting cells likely facilitates islet antigen presentation to autoreactive T cells (Figure 2A). Moreover, while antibody deposition in the islets is not a common feature of T1D, perhaps because the known islet autoantigens are not expressed on the β-cell surface, there are reports that it does occur [18]. Immunoglobulin deposition in the islets may trigger the activation of NK cells, leading to antibody-dependent cellular cytotoxicity (ADCC) and the direct killing of the β cells. In addition, higher titers of anti-insulin and anti-IA-2 autoantibodies is associated with an increased risk and rate of progression of T1D, which is consistent with a role for autoantibodies in T1D pathogenesis [19,20,21]. Additionally, higher-affinity antibodies also correlate with disease progression, implying that not only the presence but also the quality of antibodies is important in the disease progression to T1D [19,22,23]. While the exact mechanism(s) by which elevated autoantibody titers and the affinity for self-antigen are associated with a more rapid progression of T1D are unknown, it is tempting to speculate that these factors enhance autoantigen–antibody immune complex formation, which leads to FcγR-mediated activation and antigen presentation by other professional antigen-presenting cells (dendric cells and macrophages) to self-reactive T cells. High autoantibody titers and affinity for self-antigen may also be a reflection of the increased formation of autoantibody-secreting plasma cells that have undergone somatic mutations in a germinal center, indicative of an overall heightened immune response and, perhaps, more T follicular helper cells. Taken together, while islet-reactive antibodies are not sufficient or necessary to cause disease, they are likely not as benign as has been previously suggested.

### 2.3. Antigen Presentation of B Cells in T1D

A second major effector function of B cells is the antigen presentation to CD4 T cells and cross-presentation to CD8 T cells (Figure 2B). As previously mentioned, both T-cell subsets are essential to diabetogenesis. While various professional antigen-presenting cells (APCs), such as DCs and monocytes, are able to activate T cells, B cells are nearly 1000-fold more efficient at presenting their recognized antigen than non-specific APCs due to recognition by their B-cell receptor (BCR) [24,25]. Evidence for the requirement of antigen presentation by B cells to both CD4 and CD8 T cells is demonstrated by the diabetes protective effects in NOD mice when B cells are deficient in either MHC class I or II [16,26,27].

B cells are unique among antigen-presenting cells, in that they preferentially present antigen recognized by their BCR [28]. Therefore, it is not the ability to present any antigens to T cells that contributes to autoimmunity, but the ability to present autoantigens. It has been demonstrated that approximately 20% of peripheral naïve B cells in humans are autoreactive [29]. Furthermore, it has been shown that the frequency of naïve autoreactive B cells is even higher in individuals with autoimmunity such as T1D, rheumatoid arthritis (RA), or systemic lupus erythematosus (SLE) [30,31,32]. The escape of autoreactive B cells from central tolerance in the bone marrow is likely driven, in part, by genetic risk alleles. Together with the loss of the peripheral tolerance mechanism, called anergy, which has been shown to be lost in early T1D, individuals with T1D have an increased frequency of self-reactive B cells in their periphery poised to participate in disease [11,33]. Indeed, a variety of mouse models has demonstrated the importance of B-cell autoantigen specificity in the development of autoimmune diabetes. For example, in the VH125.NOD mouse, all B cells express the anti-insulin 125Tg heavy chain that is capable of rearranging with any endogenous light chain. In this model, 1–2% of peripheral B cells are insulin-specific compared to 0.1% in WT NOD. Importantly, VH125.NOD mice have an increased penetrance and accelerated rate of diabetes development compared to conventional NOD mice [34]. However, in the VH281.NOD mouse, which lacks insulin-reactive B cells in the periphery, mice are largely protected from disease [21]. Other antigen-specific B cells have also been shown to contribute to disease. Indeed, Leeth et al. demonstrated that transgenic peripherin-autoreactive B cells invade NOD pancreatic islets, adopt an activated and proliferative state, and strongly promote the rapid progression of autoimmune diabetes [35]. Lastly, while it is difficult to conclusively demonstrate that B cells are critical antigen-presenting cells in humans, recent studies have identified a population of insulin-binding B cells that expresses increased CD80/CD86 on the cell surface and is significantly increased in the pancreatic lymph node of T1D donors compared to non-diabetic donors [33]. These findings suggest that these cells can likely act as potent antigen-presenting cells to T cells. Moreover, Nicholas et al. identified that islet-reactive B cells from autoantibody positive Stage 1 or 2 and recent-onset T1D patients demonstrate an elevated expression of genes associated with antigen processing and presentation, such as HLA genes and CIITA, compared to HLA-matched non-diabetic controls [36]. Furthermore, B cells in the pancreas of patients with T1D have been found to be in close proximity to CD8 T cells, implying a possible role in activation of pancreatic T cells at the site of disease attack [18,37,38]. In summary, B-cell antigen presentation through MHC class I and II is crucial for activating self-reactive CD8+ and CD4+ T cells, respectively, which likely promotes pancreatic β-cell destruction in T1D (Figure 2B).

### 2.4. Cytokine Production by B Cells in T1D

The third effector function of B cells involves the production of cytokines (Figure 2C). It is widely accepted that the production of inflammatory cytokines is a major contributor to T1D pathogenesis; however, the contribution of cytokines derived from B cells is less clear. Nevertheless, B cells have been shown to produce a variety of cytokines, including IL-6, IL-12, TNF-α, and IFN-γ [39], which have all been implicated in the pathogenesis of T1D [40,41,42]. T1D proinflammatory cytokines, such as IFN-γ, facilitate the migration of T cells into islets and increase the recognition of β cells by CD8 T cells by increasing MHC class I on the surface of β cells [40]. TNF-α, on the other hand, primarily functions to increase the local production of IL-1β and IL-6 and activate cytotoxic and apoptotic responses (Figure 2C) [43]. Studies in T1D subjects have shown that recently activated insulin-binding B cells produce elevated levels of IL-6, IFN-γ, and TNF-α, compared to age-matched controls [33]. The secretion of IFN-γ by B cells plays a key role in regulating the development of TH1 cells, while TNF-α contributes to inflammatory responses [44]. This study highlights the potential of B cells to influence immune responses through the production of these cytokines, similar to other antigen-presenting cells, such as dendritic cells [39]. An important consideration is that the activation and skewing of T cells from B-cell cytokine production has been primarily elucidated in the context of infection. Therefore, while there is strong evidence that B cells are able to produce various inflammatory cytokines, the precise role for B-cell-derived cytokines in the context of T1D remains to be fully elucidated.

### 2.5. Regulatory B Cells in T1D

In addition to producing inflammatory cytokines, B cells can also secrete a range of anti-inflammatory cytokines, such as IL-10 and IL-35. These regulatory B cells (Bregs) are thought to play a crucial role in modulating the local immune response. Circumstantial evidence for a regulatory role for B cells in T1D stems from the observation that B-cell depletion has a paradoxical effect on T cells. While the protective effects of B-cell depletion in T1D is well established [6,7], various lab groups have reported an increase in Th1 and Th17 responses following B-cell depletion, which could be due to the loss of Breg cells and/or expansion of effector T cells following lymphodepletion [45]. Future studies are needed to address these possibilities. To date, evidence for the role of dysfunctional Breg cells in T1D remains inconclusive. Several groups have reported conflicting findings regarding numerical Breg defects in T1D [6,7,46,47,48,49]. These discrepancies are likely due to the absence of well-defined surface markers for Bregs, making consistent identification difficult. For example, Wang et al. demonstrated that the frequency of CD24^hi^CD38^hi^ Breg cells was decreased in T1D patients and produced less IL-10 compared to controls. The decreased frequency of Bregs negatively correlated with TNF-α- and IFN-γ-producing CD4+ T cells [50]. Similarly, Kleffel et al. demonstrated that IL-10-producing B cells were decreased in T1D donors compared to controls, but were overall able to suppress IFN-γ production by T cells in vitro in the presence of autoantigen [51]. In addition, IL-35+ Breg cells have been shown to be increased in long-standing T1D patients who still have detectable levels of C-peptide compared to those who have lost endogenous insulin production [52]. In summary, it is evident that B cells can produce a variety of pro- and anti-inflammatory cytokines, and that B-cell cytokine production is altered in patients with T1D. Future studies are needed to more directly demonstrate a strong mechanistic role for B-cell-derived cytokines in the pathogenesis of T1D.

## 3. B-Cell Interaction with the Pancreas

### 3.1. Islet Architecture and Why β Cells and Not α Cells

**Figure 3:**
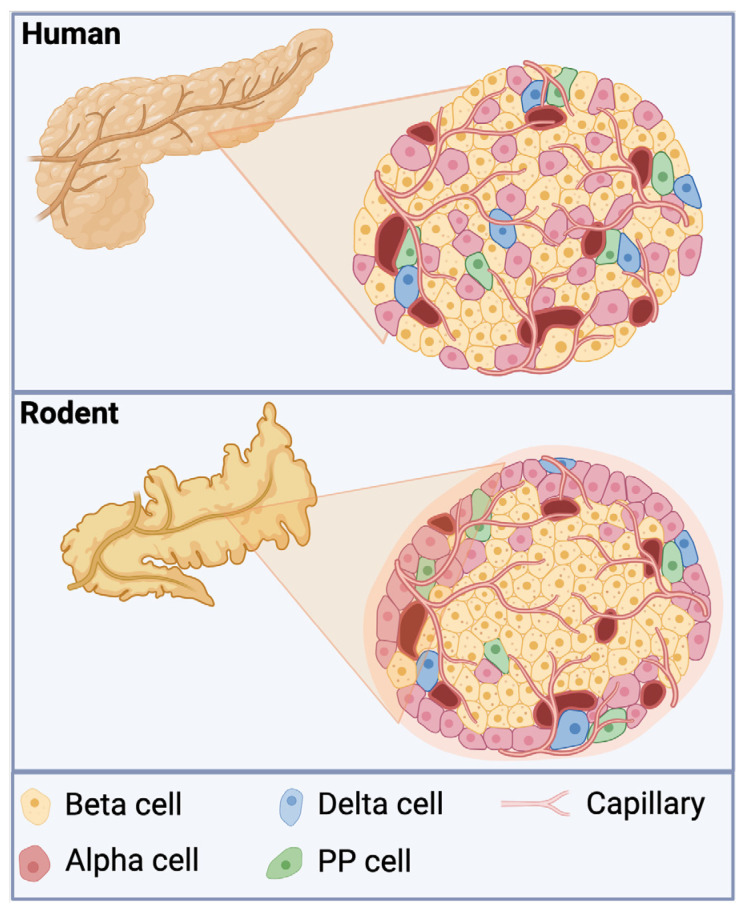
Direct comparison of human and rodent islet architecture. Human islets cell populations are intermixed among each other. Rodent islets have a core of β cells surrounded by the α, δ, and PP cells.

The pancreas has dual exocrine and endocrine functions, which have both been studied extensively [53]. The islets of Langerhans are part of the endocrine pancreas and contain insulin-producing β cells that are the target of immune attack in T1D. Islets contain several thousand endocrine cells, including alpha (α), beta (β), delta (δ), epsilon (ε), and pancreatic polypeptide (PP) cells. The majority of cells in islets are β cells, making up 50–70% and 60–80% of the islets in humans and mice, respectively [54] (Figure 3). Alpha cells are the next most abundant cell type, contributing 20–40% and 10–20% to the total number of cells in humans and mice, respectively [55,56,57,58,59]. A key difference between mouse and human islets lies in their architecture. Cells within human islets are intermixed among each other, whereas in rodent islets, the β cells form a core in the center of the islet, which are surrounded by the α, δ, and PP cells (Figure 3).

An intriguing aspect of T1D is the selective destruction of β cells by the immune system, while neighboring α cells remain largely unaffected. Differential gene expression analysis has shown that β cells are more frequently targeted due to several factors [60]. β cells have lower levels of the anti-apoptotic gene BCL2L1 and higher expression of the pro-apoptotic gene CHOP, indicating a greater susceptibility to endoplasmic reticulum stress. They also express lower levels of HSPA5, which encodes the protective chaperone BiP. In contrast, α cells express higher levels of HLA-E, which may reduce their immunogenicity. Lastly, CD8+ T cells, which are the executioners of disease, are largely reactive to β cell antigens, such as pre-proinsulin, but not to glucagon, suggesting an intrinsic immunogenicity of β cells over α cells [60]. Likewise, although autoantibodies to α cells do exist, they are less frequent and not significantly associated with T1D [61,62]. Instead, B cells also recognize islet-antigens such as insulin, GAD65, and IA-2.

### 3.2. B Cells and β-Cell Stress in the Pathogenesis of T1D

Over the past few years, it has become well accepted that β-cell stress plays a crucial role in the pathogenesis of T1D. Due to their secretory function, β cells are particularly vulnerable to endoplasmic reticulum stress [63]. For example, when activated by a stimulus, such as high glucose, β cells can increase (pro)insulin synthesis more than 10-fold [64]. Severe and prolonged stress can lead to β-cell dysfunction and death, resulting in the increased release and exposure of self-antigens. Given that the major B-cell autoantigens are all intracellular proteins found in β cells, it is likely that the continuous release of self-antigens due to ongoing β-cell stress and death sustains the activation and persistence of self-reactive B cells. As mentioned above, these B cells not only produce autoantibodies but also present self-antigens to T cells, advancing T1D pathology.

Additionally, β-cell stress can lead to the formation of neo-epitopes, which are novel protein fragments generated through post-translational modifications (PTM) or alternative splicing [65]. Among these neo-epitopes are hybrid insulin peptides (HIPs), which are formed through the covalent linkage of insulin fragments to other protein fragments [66]. Over the years, various HIP-reactive or PTM islet-reactive T cells have been found to be increased in the blood and pancreas of T1D donors compared to non-diabetic donors [66,67,68]. Furthermore, antibodies recognizing HIPs have been detected in the sera of T1D patients, indicating that these self-peptides are also recognized by B cells in the context of disease [69]. It is tempting to speculate that HIP-reactive B cells may also help drive disease pathogenesis through presentation to T cells. More studies are needed to address this possibility. Thus, the presence of these stress-induced neo-epitopes, including HIPs, in β cells, which are also recognized by autoreactive B and T cells, highlights the interplay between β-cell stress and autoimmunity in T1D.

### 3.3. Timing of B Cells into the Islets During Disease Progression

A defining feature of T1D is insulitis, which is characterized by the infiltration of immune cells into the islets, leading to the gradual destruction of insulin-producing β cells. In both the NOD mouse model and human T1D, B cells can be found infiltrating the pancreatic islets. However, in NOD mice, B-cell infiltration tends to occur earlier than in human T1D and is more pronounced. Therefore, due to the differences in islet architecture (Figure 3) and disease progression in NOD mice versus humans, findings from mouse studies may not directly correlate with human T1D. Nevertheless, as early as 1985, B cells have been found alongside CD8+ T cells in the pancreas of human donors with type 1 diabetes [18]. More recent studies utilizing human pancreatic tissue have demonstrated that CD20+ B cells appear soon after the onset of insulitis, acting as the second-largest immune-cell component after CD8+ T cells [70]. These B cells increase in accordance with the degree of β-cell destruction but sharply decrease once insulin-producing β cells are depleted [70]. Interestingly, as the number of CD8+ T cells increased, so did CD20+ B cells in the islets, which was not the case for CD4+ T cells or macrophages. This finding suggests that there is an important relationship/association between B cells and CD8+ T cells in driving islet destruction. Given that islet B cells did not appear to be plasma cells, since they lacked the expression of CD138, this relationship is likely driven by antigen cross-presentation of B cells to CD8+ T cells [31]. Closely mimicking human T1D, the NOD mouse recapitulates the pattern of lymphocyte infiltration, where T cells are the predominant lymphocyte, followed by B cells [71]. In mice, B-cell infiltration occurs as early as 2 weeks of age. Specifically, B-1a cells, a subset of innate-like B cells that predominately reside in the peritoneal cavity, have been shown to migrate to the pancreas at 2 weeks of age and even precede entry by T cells [72]. These B1-a cells can activate plasmacytoid dendritic cells through the production of anti-dsDNA antibodies, which ultimately leads to the production of IFNα and the initiation of diabetes [3,73].

Studies focused on understanding the phenotype and function of B cells in the pancreas during autoimmune diabetes in NOD mice have demonstrated that during established insulitis, the B cells take on a more follicular phenotype [72]. In addition, some of the islet-infiltrating B cells during established insulitis downregulate CD20 expression on their cell surface and upregulate the plasma cell marker, CD138 [74,75]. Overall, these findings demonstrate that the B-cell subsets that infiltrate the islets change over time, and therefore, could play varying roles in the pathogenesis of autoimmune diabetes in the NOD mouse. More studies of the B-cell phenotypes in human pancreas along a continuum of diabetes progression are needed to understand whether this same phenomenon also occurs in human T1D.

### 3.4. B Cells and Tertiary Lymphoid Structures in the Pancreas

The temporal relationship between B cells and T cells is essential for understanding their combined role in insulitis. In fact, insulitis resulting from the recruitment of B cells and T cells into the islets closely resembles that of tertiary lymphoid structures (TLS). These TLS provide an organized environment for B–T cell interactions, including T-cell zones, B-cell follicles, and germinal centers (GCs), allowing for effective antigen presentation and immune-cell activation. TLS have been found in the pancreas of both human donors and NOD mice [76,77], and one study found an inverse correlation between the frequency of islet TLS and the age of onset [77]. Hence, the more rapid progression of disease that is seen in young-onset T1D (discussed in more detail below) may be due to the increased presence of TLS in the pancreas that fuels the activation of autoreactive lymphocytes.

Much of what we know regarding the role of B cells in TLS have come from studies of cancer. For example, in some cancer models, TLS have been shown to contribute to the development of tumor-targeting immunity and are correlated with better patient survival, i.e., better activation of tumor-reactive lymphocytes. Several studies have investigated the role of B cells within TLS using B-cell depletion. Using isolated human tumor biopsies, B-cell depletion in TLS promotes signs of a dysfunctional CD8+ T-cell compartment among tumor-infiltrating lymphocytes [78]. Another study demonstrated that B-cell depletion inhibited the formation of TLS, which promoted tumor growth in a mouse cancer model [79]. Together, these results show that B cells in tumor-TLS are important for developing tumor-specific immunity. Similarly, it has been shown that NOD mice deficient in B cells are resistant to the development of insulitis and recruitment of T cells, and are protected from diabetes development [5,79,80,81], suggesting that B cells are also required for the formation of TLS in autoimmune diabetes. Therefore, the formation of TLS in the pancreas is likely necessary for the activation of autoreactive T cells that ultimately destroy the insulin-producing β cells, which is likely driven by the presence of B cells in the TLS.

### 3.5. Evidence for Endotypes in T1D

It is becoming clear from the correlation between disease severity and age that there are endotypes for T1D. More specifically, younger patients present with more B-cell infiltration into islets and experience a more rapid disease onset. The pancreatic lesions of patients under 12 years of age show a higher concentration of B cells, which is associated with more severe β-cell loss [82]. This young-onset, rapid-progressing T1D, with increased numbers of B cells in the pancreas, has been coined the “CD20+Hi” endotype. This contrasts with individuals with a “CD20+lo” profile, who tend to be older-onset T1D patients that undergo a slower progression of β cell loss. This distinct B-cell phenotype based on age of onset was supported in a different study that compared levels of C-peptide, a measurement of β-cell function, and variations in gene expression using whole blood RNA sequencing in new-onset T1D subjects. The results demonstrated that young-onset T1D subjects exhibited a rapid loss in C-peptide, which was associated with an increased expression of B-cell genes [83]. Hence, studies support the concept of different endotypes in T1D, which is, at least, partially explained by the increase presence of B cells in the pancreas of young-onset T1D subjects, who undergo a more aggressive form of disease. These findings suggest that B cells likely play a more important role in the rapid progression of T1D seen in young-onset individuals [8].

Hence, the presence of B cells is required for disease to occur in the NOD mouse [5,80,81], must be specific for islet autoantigens [5,34], and must be capable of antigen presentation via MHC class I and II [13,26]. Together, these findings illustrate the complex and dynamic role of B cells in the pathogenesis of T1D, from their early involvement in insulitis to their continued presence in advanced disease stages. Gaining a better understanding of the timing, phenotype, and interactions of B cells within the human pancreas may open new avenues for therapeutic interventions targeting specific stages of T1D progression.

## 4. B-Cell Therapies

The use of B-cell-targeted therapies has emerged as a key approach in treating autoimmune diseases, including T1D. Given that B cells play a critical role in autoimmune pathogenesis, therapies that modulate or deplete pathogenic B cells aim to mitigate the immune system’s attack on self-tissues without broadly suppressing immunity. In this section, we give an overview of existing and novel B cell depletion strategies, highlighting their mechanisms, efficacy, and limitations.

### 4.1. Established Antigen-Agnostic B-Cell-Depleting Agents

Anti-CD20 therapies, such as rituximab, represent a foundational strategy for B-cell depletion. Targeting CD20, a protein uniquely expressed on the surface of B cells, this monoclonal antibody therapy binds to B cells, leading to their destruction via ADCC or complement dependent cytotoxicity (CDC). In the NOD mouse model, treatment with anti-CD20 antibody in early stages prevents the development of autoimmune diabetes [84]. In addition, treatment after disease onset also reversed diabetes in mice that had already developed hyperglycemia, highlighting the potential of CD20-targeted therapies in both the prevention and reversal of T1D. While rituximab effectively depletes mature B cells, but not plasma cells, it does not correct defects in early B-cell tolerance checkpoints, which can result in a resurgence of autoreactive B cells [75,85]. Serreze et al. highlighted the challenge of CD20-targeted therapies in T1D by showing that not all pathogenic B cells express CD20 [74]. In their study, they observed that intra-islet B cells responsible for promoting β-cell destruction in T1D often lose CD20 expression during the disease process. This limitation is particularly noted in T1D, where a subset of autoreactive B cells may evade anti-CD20 treatment. 

An alternative strategy targets CD22, another B-cell surface molecule that can act as a negative regulator of B-cell signaling. Unlike anti-CD20, anti-CD22 therapy has been shown to reprogram B cells in diabetic mice [86]. This treatment reversed established T1D by shifting the B-cell profile towards a more tolerogenic state, indicating that CD22-targeted therapies might be effective not only in preventing disease but also in inducing remission.

**Figure 4:**
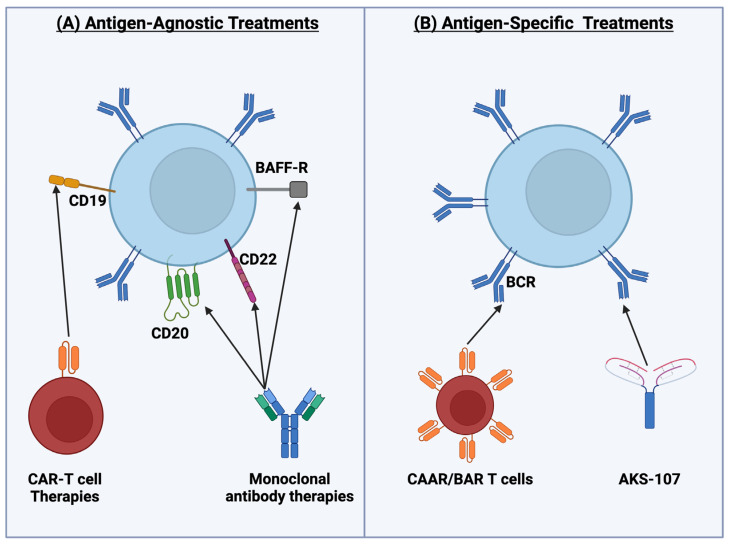
B-cell depletion therapies. (**A**) Antigen-agnostic B-cell depletion therapies; (**B**) antigen-specific B-cell depletion therapies.

Beyond surface antigen targeting, therapies like belimumab inhibit B-cell survival by blocking the B-cell activating factor (BAFF). This monoclonal antibody is currently approved for the treatment of non-renal SLE. It binds soluble BAFF, which obstructs its contact with BAFF-R, TACI, and BCMA receptors on the surface of B cells, thereby restricting B-cell receipt of the pro-survival factor [87]. Similarly, others have investigated direct blockage of the BAFF receptor (BAFFR) using a BAFFR-Fc monotherapy in NOD mice. They found that continuous treatment with BAFFR-Fc significantly inhibited the development of autoimmune diabetes, regardless of the presence of insulin autoantibodies prior to treatment [88] (Figure 4A).

### 4.2. Next-Generation Antigen-Agnostic B-Cell Therapies

Innovative therapies, such as CAR-T cell treatment, represent a transformative approach to B-cell depletion. CAR-T cells are engineered to recognize specific B-cell antigens, enabling precise and potent cytotoxic responses (Figure 4A). CD19 CAR-T-cell therapy has emerged as a promising approach for treating autoimmune diseases, including SLE and T1D, due to its ability to deplete a broader range of B cells and better target tissue-resident B cells compared to traditional therapies like anti-CD20 monoclonal antibodies [89]. Another CAR-T cell, 287-CAR CD8+ T cell, was recently developed to target the insulin B chain peptide 9:23, a fragment known to be presented on MHC class II that is important in development of disease, in the context of MHC class II on antigen-presenting cells, such as B cells [90]. Treatment with 287-CAR CD8+ T cells decreased the incidence of autoimmune diabetes in NOD mice. Since diabetes development requires antigen presentation by B cells, it is likely that the 287-CAR-T cell acts, in part, by targeting insulin-reactive B cells that are presenting antigen to pathogenic T cells [91].

Newer generations of monoclonal antibodies targeting CD20 have also been developed, such as ocrelizumab [92]. A key difference between ocrelizumab and rituximab lies in their design. Ocrelizumab is a humanized monoclonal antibody, and rituximab is a chimeric monoclonal antibody, making the former less likely to cause an anti-drug immune response. Interestingly, ocrelizumab has an epitope different to but overlapping that of rituximab and is thought to bind more effectively to CD20, potentially leading to the more efficient depletion of B cells [93].

### 4.3. Antigen-Specific B-Cell Depletion

Researchers have continued to strive to make treatments more precise, and therefore, a need for antigen-specific depletion therapies have been developed (Figure 4B). By focusing on B cells reactive to autoantigens, such therapies minimize off-target effects and spare non-autoreactive B cells. For example, Henry et al. explored insulin-specific B-cell depletion by administering a monoclonal antibody that recognizes insulin and found that this treatment depleted insulin-binding B cells and prevented disease in NOD mice [94]. Another antigen-specific depletion therapeutic, AKS-107, is a novel Insulin-Fc fusion immunotherapeutic that targets insulin-specific B cells. This therapy selectively deletes insulin-reactive B cells, effectively preventing the onset of autoimmune diabetes in mouse models [94,95]. The use of antigen-specific targeted approaches could have widespread applications across autoimmune diseases where specific autoreactive B-cell subsets drive pathology.

More recently, “BAR” (B-cell-targeting antibody receptor) Tregs and “CAAR” (chimeric autoantibody receptor) T cells have been shown to prevent antigen-specific antibody production by inducing tolerance or depleting self-reactive B cells [91,96,97,98]. Broadly, these cell-based therapies express fusion receptors where one end has an autoantigen, and the other end is composed of an intracellular signaling domain. When the cell-based therapies encounter the antigen-reactive B cells by virtue of BCR recognition to their self-antigen expressed on the BAR/CAAR, depletion is triggered. Once these therapies are approved for treatment, they will launch a new era of available therapies for human autoimmune diseases.

### 4.4. Combination Therapies

Combining B-cell depletion with other immunomodulatory agents represents an exciting frontier in therapy optimization. For instance, pairing anti-CD20 with BAFF blockage has demonstrated a synergistic effect on the NOD mouse [88]. While human trials have yet to explore this combination fully in T1D, its potential is supported by evidence from SLE studies showing improved outcomes with dual therapy, emphasizing the value of multi-targeted approaches in managing complex autoimmune conditions. There is a current clinical trial investigating the use of rituximab and abatacept for the treatment of T1D. This combination depletes B cells (via anti-CD20 antibodies) and inhibits T cell activation (CTLA-4Ig). It may be possible, in the future, to test other combinations, such as depleting/anergizing both B and T cells together with teplizumab and rituximab, or by depleting B cells and preserving β-cell function using rituximab and verapamil.

Overall, B-cell depletion therapies have broadened the scope of autoimmune treatment by offering selective and potentially reversible immune modulation. While anti-CD20 therapies have set a precedent, challenges in addressing specific autoreactive B-cell subsets underscore the need for next-generation strategies. Antigen-specific therapies, CD22 targeting, CAR-T cell approaches, and combination therapies are paving the way for more precise and effective treatments that may transform the management of autoimmune diseases like T1D. Further research into B-cell phenotypes, their role within affected tissues, and the optimal timing of intervention will be essential for advancing these therapies toward clinical success. 

## 5. Conclusions

In summary, B cells play a multifaceted role in the pathogenesis of T1D, acting as crucial intermediaries in the islet–immune-cell interface. They contribute to disease progression through antigen presentation, cytokine production, and antibody secretion, which, collectively, can activate autoreactive T cells and potentiate pancreatic inflammation. In addition, B cells form unique structures in and around the islets and correspond with specific disease endotypes based on the age of onset. Hence, the complexity of T1D necessitates further exploration of B-cell functions and their therapeutic potential. Current and emerging B-cell-targeted therapies, including anti-CD20, CAR-T cells, and antigen-specific approaches, offer promising avenues for modulating the immune response and potentially reversing disease progression. Continued research into the timing, phenotype, and interactions of B cells within the pancreas is essential for developing effective interventions that could transform the management of T1D.

## Data Availability

Not applicable.

## Abbreviations

The following abbreviations are used in this manuscript:

T1D	Type 1 diabetes
NOD	Non-obese diabetic mouse model
WT	Wild type
DC	Dendritic cell
NK	Natural killer cell
ADCC	Antibody-dependent cellular cytotoxicity
APC	Antigen-presenting cells
BCR	B-cell receptor
RA	Rheumatoid arthritis
SLE	Systemic lupus erythematosus
Bregs	Regulatory B cells
PTM	Post-translational modification
HIP	Hybrid insulin peptide
TLS	Tertiary lymphoid structure
GC	Germinal center
CDC	Complement-dependent cytotoxicity
BAFF	B-cell activating factor
BAFFR	BAFF receptor
BAR	B-cell-targeting antibody receptor
CAAR	chimeric autoantibody receptor
